# Genomic Profiling in Glioma Patients to Explore Clinically Relevant Markers

**DOI:** 10.3390/ijms252313004

**Published:** 2024-12-03

**Authors:** Viacheslav Varachev, Olga Susova, Alexei Mitrofanov, David Naskhletashvili, George Krasnov, Anna Ikonnikova, Svetlana Bezhanova, Vera Semenova, Nadezhda Sevyan, Evgenii Prozorenko, Yulia Ammour, Ali Bekyashev, Tatiana Nasedkina

**Affiliations:** 1 Engelhardt Institute of Molecular Biology, Russian Academy of Sciences, 119991 Moscow, Russia; varachevviacheslav95@mail.ru (V.V.); gskrasnov@mail.ru (G.K.); anyuik@gmail.com (A.I.); sulpiridum@yandex.ru (V.S.); 2 N.N. Blokhin Russian Cancer Research Center of the Ministry of Health of the Russian Federation, 115478 Moscow, Russia; susovaolga@gmail.com (O.S.); mitrofanov-aa@list.ru (A.M.); nas-david@yandex.ru (D.N.); dmitrownaja@gmail.com (S.B.); hope.sev@ya.ru (N.S.); prozorenko1984@mail.ru (E.P.); abekyashev@gmail.com (A.B.); 3 I.I. Mechnikov Research Institute for Vaccines and Sera, 105064 Moscow, Russia; yulia.ammour@yahoo.fr

**Keywords:** glioma, glioblastoma, recurrence, multigene sequencing, mutations, copy number variation, survival, targeted treatment

## Abstract

Gliomas are a heterogeneous group of brain tumors, among which the most aggressive subtype is glioblastoma, accounting for 60% of cases in adults. Available systemic treatment options are few and ineffective, so new approaches to therapies for glioblastoma are in high demand. In total, 131 patients with diffuse glioma were studied. Paired tumor–normal samples were sequenced on the Illumina platform; the panel included 812 genes associated with cancer development. Molecular profiles in clinically distinct groups were investigated. In low-grade glioma (LGG) patients (n = 18), the most common mutations were *IDH1/2* (78%), *ATRX* (33%), *TP53* (44%), *PIK3CA* (17%), and co-deletion 1p/19q (22%). In high-grade glioma (HGG) patients (n = 113), more frequently affected genes were *CDKN2A/B* (33%), *TERTp* (71%), *PTEN* (60%), *TP53* (27%), and *EGFR* (40%). The independent predictors of better prognosis were tumor grade and *IDH1/2* mutations. In *IDH*—wildtype glioblastoma patients, a history of other precedent cancer was associated with worse overall survival (OS), while re-operation and bevacizumab therapy increased OS. Also, among genetic alterations, *TERTp* mutation and *PTEN* deletion were markers of poor prognosis. Nine patients received molecular targeted therapy, and the results were evaluated. The search for molecular changes associated with tumor growth and progression is important for diagnosis and choice of therapy.

## 1. Introduction

Gliomas are primary diffuse lesions of the brain involving cerebral structures and are the most common neoplasms of the central nervous system (CNS). Gliomas can develop at any age, but are most common in the elderly [1,2]. Since gliomas are an extremely heterogeneous group of tumors, accurate classification is very important, as it largely determines the prognosis and treatment of patients. Traditionally, two main categories of gliomas have been distinguished: diffuse gliomas and non-diffuse gliomas. Diffuse gliomas are characterized by migration of tumor cells over long distances into the CNS parenchyma, which excludes the possibility of complete surgical resection. In contrast to diffuse gliomas, non-diffuse gliomas tend to be more circumscribed. Diffuse gliomas are commonly thought to originate from neuroglial progenitor cells, and histologic subtypes (astrocytoma, oligodendroglioma, and glioblastoma) are distinguished on the basis of morphologic similarity to neuroglial cells found in normal tissue [3]. The grade of malignancy is assigned based on the presence or absence of prominent mitotic activity, necrosis, and/or abundant microvascular proliferation [3,4]. Grade I gliomas are the slowest growing and are generally considered borderline between benign and malignant neoplasm, while grade II, III, and IV gliomas are classified as malignant tumors. The most common malignant grade IV glioma with a highly aggressive course is glioblastoma, accounting for 60% of all adult brain tumors [5,6,7]. Glioblastoma has the least favorable prognosis and even with intensive treatment, including radiation therapy and chemotherapy with temozolomide after the first surgery, the risk of recurrence or progression remains very high [8,9,10]. Systemic treatment standards for patients with recurrent glioblastoma are poorly defined, and the available options are few and ineffective [11,12,13]. The median life expectancy of glioblastoma patients under 70 years of age is about 14.6 months, and only 3–5% of patients live longer than three to five years after diagnosis [14,15].

The 2007 WHO classification was purely morphological, it did not use genetic characteristics to divide tumors into groups [3]. Further investigation of tumor biology has made it possible to identify key genetic alterations that can define the molecular pattern of tumor development and discover targets that can be used for diagnostic, prognostic, or therapeutic purposes [16,17,18]. Point mutations in isocitrate dehydrogenase 1 and 2 (*IDH1/IDH2*) genes identified in diffuse gliomas with different degrees of malignancy have become the main factor for molecular-based classification of gliomas. The significant difference in survival in *IDH*-mutation carriers reflects its high prognostic value for these tumors [19,20]. The second molecular event underlying the classification of gliomas is the co-deletion of 1p19q, which occurs predominantly in oligodendrogliomas with a frequency of 75–80%, and is associated with a favorable clinical course of the disease and high efficacy of radiation and chemotherapy [21]. Glioblastoma has also received comprehensive molecular characterization as part of The Cancer Genome Atlas (TCGA) project [22]. The genetic lesions inherent in glioblastoma have been shown to affect telomere length, chromatin regulation, and intracellular signaling, involving receptor tyrosine kinases (epidermal growth factor receptor (EGFR), FGFR, BRAF), mitogen-activated protein kinase, and phosphoinositide-3-kinase (PI3K/AKT/mTOR), as well as the retinoblastoma/E2F and the p53 tumor suppressor pathways [23,24,25]. The 2016 WHO classification of CNS tumors (2016 WHO CNS) was the first classification that combined morphological and genetic characteristics of tumors [26]. Diffuse gliomas, including glioblastoma, were divided into three subtypes: *IDH*—mutant, *IDH*—wildtype, and *IDH*—NOS (not otherwise specified). The proposed glioma diagnostic algorithm involved a simple scheme of step-by-step detection of *IDH* mutation and 1p19q co-deletion in combination with already established tumor morphology. At the same time, the simultaneous use of histological and genetic features in tumor diagnosis led to controversial statements in some cases.

The last revision resulted in the 2021 WHO classification, fifth edition (2021 WHO CNS), which prioritized known molecular parameters to refine the diagnosis of CNS tumors, and particularly, diffuse gliomas. The key diagnostic criteria for diffuse gliomas of adults are mutations in isocitrate dehydrogenase genes *IDH1* and *IDH2*; other primary genetic markers include co-deletion 1p/19q, nuclear alpha-thalassemia/mental retardation X-linked syndrome (*ATRX*) gene mutations, loss of cyclin-dependent kinase inhibitor 2A (*CDKN2A*), *EGFR* amplification, combined gain of chromosome (chr) 7 and loss of chr 10 (7+/10−), and telomerase reverse transcriptase (*TERT*) promoter mutations [27]. Adult-type diffuse gliomas are divided into three subtypes: (1) *IDH*—mutant astrocytoma (Grade 2, 3, 4); (2) *IDH*—mutant and 1p19q co-deleted oligodendroglioma (Grade 2, 3); and (3) *IDH*—wildtype glioblastoma (Grade 4). The previously named “*IDH*—mutant secondary glioblastoma” has been reclassified as *IDH*—mutant Grade 4 astrocytoma, which has a survival rate at least two-fold higher than *IDH*—wildtype glioblastoma. In the 2021 WHO CNS, glioblastoma is defined as a tumor with obligatory wildtype *IDH1/2*, characterized by diffuse astrocytic histology and at least one of the following features: necrosis, microvascular proliferation, gain of chr 7 and loss of chr 10, *EGFR* amplification, or *TERT* mutation [27]. The inclusion of molecular criteria allows the diagnosis of *IDH*—wildtype glioblastoma even if high-grade histopathological features, necrosis, or microvascular proliferation are not detected [28]. Integrated studies in genomics, transcriptomics, and proteomics may further refine this classification by exploring the diversity of pathologies that comprise the diffuse glioma group.

Recently, next-generation sequencing (NGS) has been increasingly used in oncology clinical practice and particularly, in neuro-oncology. This approach makes it possible to identify changes in hundreds of cancer-related genes and identify subgroups of patients who may respond to personalized targeted treatment [29,30,31]. Despite the in-depth knowledge of glioma biology, data on the clinical utility of NGS, efficacy, and feasibility of targeted therapy for patients with glioblastoma are limited. A comprehensive panel of genes specific to glioma patients has not yet been identified or made available, although active attempts have been made in this direction [32,33,34,35]. Several studies have been conducted to identify potentially significant molecular alterations via NGS panel testing and to translate the results into personalized patient management [36,37]. It is also crucial to study the interaction between molecular markers and clinical features of the patient to identify novel biomarkers of prognosis and treatment efficacy. The search for molecular alterations associated with tumor development and progression may be important for further development of precision medicine approaches to the treatment of the disease.

Our study aimed to investigate genomic landscape in different subgroups of adult diffuse glioma treated at a single institution and to understand the clinical implications and association with clinical characteristics. We also present our own experience with the application of targeted therapy based on the identification of actionable molecular alterations.

## 2. Results

### 2.1. Clinical Characteristics of Glioma Patients

Pairwise blood–tumor samples of 131 patients were collected and investigated. The clinical characteristics of the patients are given in Table 1. Among 131 patients with glioma, 69 were males and 62 females, and the median age was 58 (range from 18 to 78). Diagnosis was made according to WHO 2021 classification: 21/131 (16%) patients had Grade 2 or 3 tumors (low-grade glioma or LGG, including diffuse astrocytoma and oligodendroglioma), and 113/131 (84%) had Grade 4 tumors (high-grade tumor, HGG, diffuse astrocytoma, and glioblastoma).

In 48/131 (36.5%) of patients, two or more lobes of the brain were involved in the neoplastic process. Among unilateral tumor localizations, the temporal and frontal lobes were more often affected, in 37/131 (28%) and 36/131 (27%) of patients, respectively. A total of 83/131 (63%) patients had primary glioma, and 49/131 (37%) patients were operated on for a second or third time due to intense tumor growth. Most patients (90%) had only CNS tumors, while 13/131 (10%) of patients with glioblastoma had another previously developed non-CNS tumor and glioblastoma had thus developed as second primary malignancy (subsequent primary glioblastoma, SPGB).

Most of the patients (127/131 or 96.2%) received combined radio- and chemotherapy with temozolomide at the first step of treatment. Bevacizumab therapy (monotherapy or in combination with other drugs) was delivered to 75/131 (58%) patients. By the end of the study (August 2024), 81/131 (62%) of patients had died and 50/131 (38%) were alive; overall survival of more than 36 months was recorded in 33/131 (25%) of patients.

### 2.2. Genomic Landscape in LGG and HGG Groups of Patients

**Figure 1:**
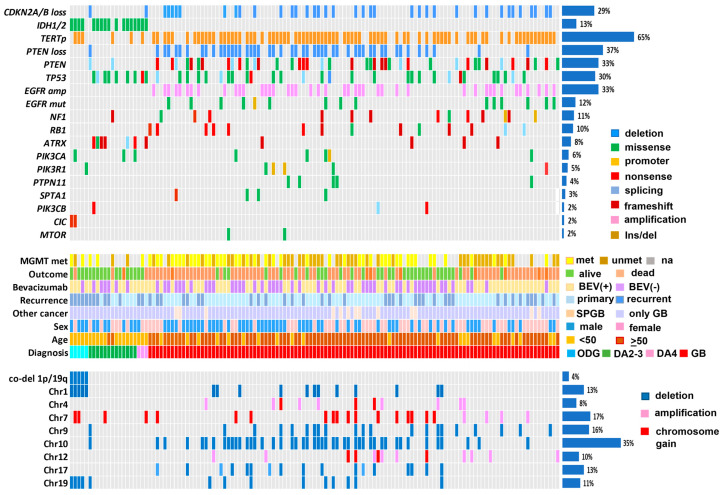
Genomic landscape of low-grade glioma (LGG) and high-grade glioma (HGG) patients. (amp—amplification, mut—mutation, met—methylated, unmet—unmethylated, na—not applicable, BEV(+)—patients received bevacizumab, BEV(-)—patients not receiving bevacizumab, SPGB—second primary glioblastoma, GB—glioblastoma, ODG—oligodendroglioma, DA2-3—diffuse astrocytoma Grade 2-3, DA4—diffuse astrocytoma Grade 4, co-del—co-deletion, chr—chromosome) (Table S1).

To analyze somatic mutations in glioma, we performed sequencing of fresh-frozen or FFPE tumor samples and matched blood samples. The genomic landscape of LGG and HGG patients is presented in Figure 1. In the total sample of 131 patients, *CDKN2A/B* loss was found in 38 (29%) cases, mutations in *IDH1/2* in 17 (13%), mutations in *TERT* gene promoter in 85 (65%), deletion of *PTEN* locus in 48 (37%), mutations in *PTEN* in 43 (33%), mutations in *TP53* in 39 (30%), *EGFR* amplification in 43 (33%), mutations in *EGFR* in 15 (12%), mutations in *NF1* in 14 (11%), *RB1* in 13 (10%), *ATRX* in 11 (8%), *PIK3CA* in 8 (6%), *PIK3R1* in 6 (5%), *PTPN11* in 5 (4%), *SPTA1* in 4 (3%), *PIK3CB* in 3 (2%), *ATM* in 3 (2%) cases, *CIC* in 2 (2%), and *MTOR* mutations in 2 (2%). Also, single mutations were revealed in the *MRE11*, *BCOR*, *BDNF*, *STAT3*, *MLH1*, *MSH6*, *PMS2*, *FGFR1*, *PDGFRA*, *DNTM3A*, *NOTCH2*, *DAXX*, *REL*, *CREBBP*, *TSC1*, *FLT1*, and *SETD2* genes (Table S1).

**Figure 2:**
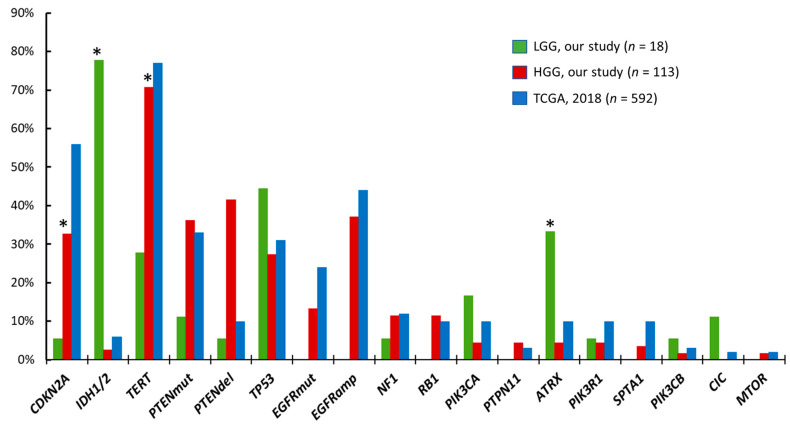
Frequency of genetic alterations in patients with LGG and HGG in comparison with data from public databases (cBioPortal, Glioblastoma multiforme TCGA PanCancer Atlas 2018). Significant difference between LGG and HGG is marked by (*).

We compared the mutational profiles of LGG and HGG patients in our cohort (Figure 2). These two samples shared the majority of mutated genes (*CDKN2A* deletion, *IDH1/2*, *PTEN*, *TP53*, *NF1*, *PIK3CA*, *PIK3R1*, *ATRX*, *PIK3CB*), but the frequencies differed substantially. Mutations in *IDH1/2* genes were much more common in LGG samples compared with HGG samples (78% vs. 3%. *p* < 0.0001), as were mutations in the *ATRX* gene (33% vs. 4%. *p* < 0.001), while *TERTp* mutations and *CDKN2A/B* deletion were more frequent in HGG samples than in LGG samples (71% vs. 28%, *p* < 0.001 and 33% vs. 6%, *p* = 0.03. respectively) (Table S2, Figure 2). Mutations in the *EGFR* gene as well as *EGFR* amplification were found only in HGG samples.

The LGG group included patients diagnosed with oligodendroglioma (ODG) (n = 7) and diffuse astrocytoma (DA), Grade 2–3 (n = 11). In ODG patients, 1p/19q co-deletion was found in five out of seven (71%), and R132H *IDH1* mutation in six out of seven (86%) patients. One patient had no *IDH1/2* mutations, but carried a 1p deletion and a mutation in the *PIK3R1* gene. Mutations in the *CIC* gene were revealed in two out of seven (29%) ODG patients. In DA Grade 2–3 patients, mutations in R132H *IDH1* were found in 7/11 (64%), and one patient had a rare R172S mutation in the *IDH2* gene. Three LGG patients were *IDH*—wildtype; one patient carried *TRIM33–NTRK2* fusion; two other patients had genotypes more specific for HGG tumor types (*CDKN2A* deletion, *PTEN* mutation, and *PTEN* deletion in one case and *TERTp* mutation, *PTEN* mutation, and *NF1* mutation in another).

In the HGG group, three patients carried R132H *IDH1* mutations and were diagnosed with DA, Grade 4. The other 110/113 (97%) HGG patients had *IDH*—wildtype glioblastoma. Comparing our HGG sample with available data from 592 glioblastoma multiforme TCGA PanCancer samples (https://www.cbioportal.org), there were no significant differences in mutation frequencies in the major affected genes (Figure 2, Table S2).

Also, recurrent chromosomal rearrangements were determined using beta-allele frequency (BAF) analysis in paired tumor–blood samples (Figure S1). Gain of chromosome 7 (or large chromosome regions including p or q arms) was found across all samples (3/18 or 17% in LGG, 19/113 or 17% in HGG). Partial or entire deletion of chromosomes 1 and 19 prevailed in LGG patients, while other rearrangements were concentrated in the HGG group. The most frequent rearrangements in this group were entire or partial loss of chromosome 10 (45/113 or 40%), chromosome 9 (20/113 or 18%), chromosome 1 (12/113 or 11%), 17 (13/113 or 12%), and 19 (10/113 or 9%). Also, recurrent CNVs occurred in the following regions: amplification of 4q12 (11/113 or 10%) (*KIT*, *PDGFA*, and *KDR* genes), amplification of 12q13 (4/113 or 3.5%) (*CDK4* gene) and 12q15 (5/113 or 4.4%) (*MDM2* gene), deletion of 17p13 (3/113 or 3%) (*TP53* gene) (Figure 2, Table S2).

The tumor mutational burden (TMB) varied from 0 to 18 mutations per megabase (muts/Mb); LGG samples harbored a mean TMB of 2.2 muts/Mb, and HGG had a mean TMB of 4.5 muts/Mb. One HGG sample had two *MSH6* mutations (W50* and 3647-1G>A) and TMB of 82 muts/Mb. The mean number of alterations in clinically relevant genes per patient (Figure 1, upper part) was slightly lower in LGG compared with HGG samples (2.4 mutations vs. 3.1 mutations), but not significantly. Genetic alterations in the PI3K-Akt-mTOR pathway (not including *EGFR* mutation or amplification) were found in 97/131 (76%) cases in the entire sample, and they were more frequent in HGG compared with LGG samples (90/113 or 79% vs. 7/18 or 38%, *p* = 0.0007). Particularly in HGG *IDH*—wildtype glioblastoma, alterations in the PI3K-Akt-mTOR pathway were discovered in 80% of samples.

Among clinically relevant variants in the *PTEN* gene (n = 43), missense mutations were revealed in 14 (32.5%) cases, nonsense in 11 (25.5%), and frameshift and splice site mutations in 9 (21%) cases in total. In 23/43 (53.4%) samples, mutations in *PTEN* were found together with *PTEN* deletion. Variants in the *TP53* gene were predominantly represented by missense mutations (28/39 or 72%), while splice site mutations (7/39 or 20%) and nonsense mutations (2/39 or 5%) were less frequent. *EGFR* mutations were missense mutations (14/15 or 93%), except for one in-frame insertion (1/15 or 7%), and were accompanied by *EGFR* amplification in 80% of cases.

### 2.3. Mutational Profiles of Primary and Recurrent Glioblastoma

Among 110 glioblastoma samples analyzed, 71 (65%) were obtained from first surgical resection (primary glioblastoma) and 39 (35%) from second or third resection (recurrent glioblastoma). Median remission between first and second resection was 10 months (range from 3 to 89 months). Mutational profiles were compared between primary and recurrent glioblastoma tumor samples, and the incidence of clinically relevant molecular alterations in both groups is presented in (Table 2).

We observed a significantly higher frequency of *TERTp* mutation, *EGFR* amplification, and *EGFR* rearrangement (including mutations and amplification) in primary glioblastoma samples compared with recurrent cases from subsequent resections (77.0% vs. 56.4%, 47.9% vs. 20.5%, and 68% vs. 25.6%, respectively. *p* < 0.05). No significant difference was found in mutation frequencies for other genes included in the analysis.

### 2.4. Clinical and Genetic Characteristics of Patients with Second Primary Glioblastoma

In a total sample of 131 patients with glioma, 13 had another cancer that preceded the development of a brain tumor. All cases were presented by so-called second primary glioblastoma (SPGB), *IDH*—wildtype. The detailed characteristics of the patients are given in Table 3.

The most common first tumor was colorectal cancer (4/13 or 30%), followed by breast cancer (3/13 or 23%), bladder cancer (2/13 or 15%), and single cases of melanoma, renal cancer, thyroid cancer, and myeloma. Median time between first tumor and glioblastoma development was 2 years (ranging from 0 to 18 years). Two patients had recurrent SPGB with an average 17.5 months of remission between first and second surgery. A patient with renal cancer and recurrent SPGB carried an *ATM* nonsense mutation and was still alive at the moment of last observation. Somatic mutations in other SPGB were characteristic of glioblastoma.

### 2.5. Survival Analysis and Cox Proportional Hazard Model

We analyzed survival rates in the whole cohort of patients with glioma and separately in the cohort of patients with HGG *IDH*—wildtype glioblastoma.

#### 2.5.1. Glioma Patients (LGG and HGG Groups)

For the whole group of glioma patients, median follow-up was 18.8 months, and median overall survival (OS) was 27.1 months (95%CI, 19.5–38). During the 1st year, the survival rate was 0.772 (95%CI, 0.701–0.850), in the 2nd year it declined to 0.550 (95%CI, 0.466–0.649), and it further dropped to 0.252 (95%CI, 0.173–0.366) by 5 years. We further evaluated the impact of known prognostic factors such as tumor grade and *IDH* mutation status on OS.

**Figure 3:**
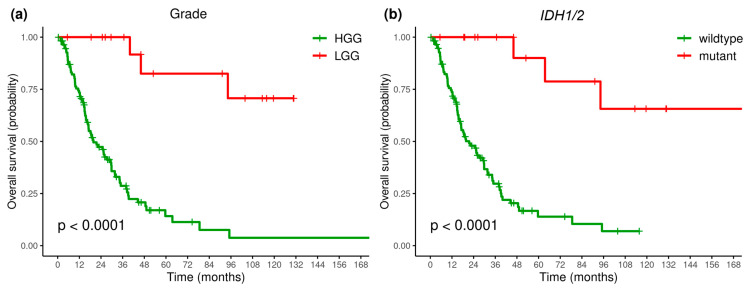
Overall survival rate depending on tumor grade (**a**) and *IDH1/2* mutational status (**b**). HGG: high-grade glioma, LGG: low:grade glioma.

The OS differed significantly between LGG and HGG patients (Figure 3a), and between *IDH*—mutant and *IDH*—wildtype patients (Figure 3b). LGG and *IDH*—mutant were associated with a more favorable prognosis, and the median OS in these patient groups was not reached, while in the HGG and *IDH*-wildtype patient groups, it was 19.78 (95%CI, 16.66–29.66) and 21.52 (95%CI, 16.66–29.8) months, respectively.

*IDH1/2* mutations were much more common in LGG patients (78%) than in HGG patients (3%), so a correlation of tumor grade and the presence of *IDH1/2* mutations was observed. However, grade and *IDH* status were significant predictors in both the univariate and multivariate Cox regression analysis, allowing them to be considered as independent prognostic factors (Table 4).

Age was significantly associated with survival in the entire study group. The HR for age was 1.04 (*p* < 0.001), indicating that each additional year of age increased the hazard of shorter length of overall survival by 4%. Meanwhile, LGG patients were significantly younger than HGG patients (median age 36.5 vs. 59 years); the HR for age adjusted by grade and *IDH*-status was 1.01 (95%CI, 0.99–1.04, *p* = 0.1869). It was concluded that age had no independent effect, due specifically to the fact that younger age was associated with more favorable subtypes.

#### 2.5.2. Survival Analysis in the HGG-*IDH*-Wildtype (Glioblastoma) Patient Group

We further analyzed OS according to mutational profile for patients with glioblastoma as a more homogeneous group, to exclude strong prognostic factors such as disease grade and the presence of *IDH1/2* mutation. Median OS in the group of glioblastoma patients was 19.5 months (95%CI, 16.1–29.6); and 1, 2, and 5-year OS were 0.726 (95%CI 0.645–0.818), 0.457 (95%CI 0.367–0.569), and 0.107 (95%CI, 0.047–0.245), respectively.

**Figure 4:**
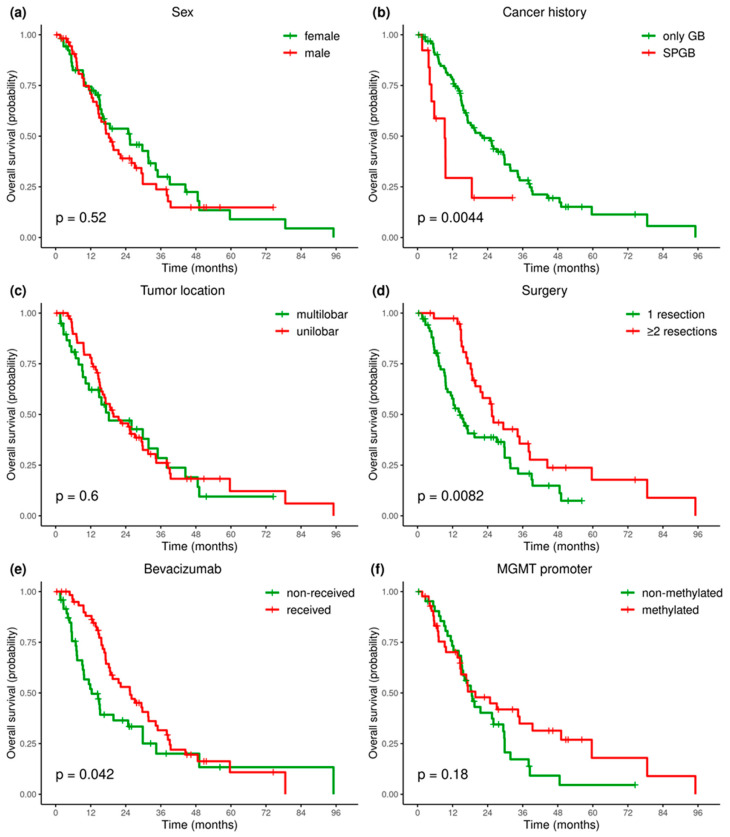
Impact of clinical features on overall survival (months) in patients with IDH—wildtype glioblastoma: age (**a**), cancer history (**b**), tumor location (**c**), number of surgical resections (**d**), treatment with bevacizumab (**e**), MGMT promoter methylation (**f**).

**Figure 5:**
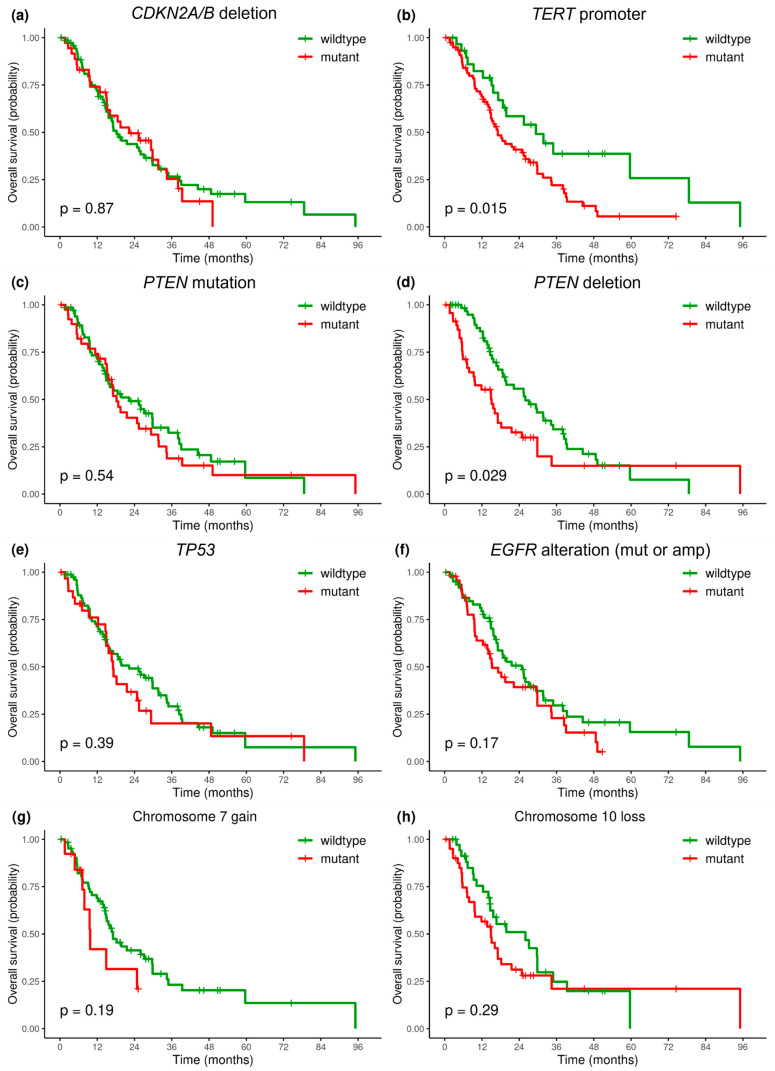
Impact of gene alteration on overall survival (months) in patients with *IDH*—wildtype glioblastoma: *CDKN2A/B* deletion (**a**), *TERT* promoter mutation (**b**), *PTEN* mutation (**c**), *PTEN* deletion (**d**), *TP53* mutation (**e**), *EGFR* alteration (mutation or amplification) (**f**), chromosome 7 gain (**g**), and chromosome 10 loss (**h**).

Kaplan–Meier curves were obtained (Figure 4 and Figure 5) and the survival analysis was fitted into a Cox proportional hazard model with the data variables of age, sex, presence of other cancer, number of surgical resections, tumor location, bevacizumab treatment, and *MGMT* promoter methylation status. Also, genetic alterations in *CDKN2A/B*, *TERT*, *PTEN*, *TP53*, and *EGFR* genes as well as gain of chromosome 7 and loss of chromosome 10 were evaluated (Table 5). First, the above predictors were assessed via univariate analysis.

Among clinical factors, precedent history of other cancer, recurrent surgery, and bevacizumab therapy were significant predictors. Precedent history of other cancer was a poor prognostic factor, with HR = 2.7 (95% CI, 1.32–5.52, *p* = 0.006), and median OS was 9.3 vs. 22.2 months in patients with SPGB compared with patients who had glioblastoma as their only tumor, respectively (*p* = 0.0044). Patients who had undergone two or more surgical resections due to recurrent glioblastoma demonstrated better OS: HR = 0.53 (95% CI 0.32–0.85, *p* = 0.009) and median OS 25.5 vs. 15.7 months, compared with those who undergone only one operation (*p* = 0.0082). Also, patients treated with bevacizumab had better OS: HR = 0.62 (95% CI: 0.39–0.98) (*p* = 0.04) and median OS 25.5 months vs. 12.3 months compared with patients not receiving bevacizumab (*p* = 0.042). No significant differences in median survival were found depending on sex, tumor location (multilobar or unilobar glioblastoma), or *MGMT* promoter methylation status (Figure 4, Table 5 and Table S3).

We analyzed the association of OS with mutations in the most frequently affected genes: *CDKN2A*, *TERT*, *PTEN*, *TP53*, and *EGFR*. Glioblastoma patients with *TERTp* mutations in the tumor sample had worse OS compared with *TERTp* wildtype tumors: HR = 1.98 (*p* = 0.02) and median OS 17.0 vs. 29.3 months (*p* = 0.015). Also, patients with impaired *PTEN* genes due to mutation or deletion (*PTEN* alteration) had worse OS compared with wildtype *PTEN*: HR = 1.7 (*p* = 0.03), and median OS 16.1 vs. 27.1 (*p* = 0.028). Almost the same effect was observed separately for *PTEN* deletion, with HR = 1.66 (*p* = 0.03), but not for *PTEN* mutations. No significant association was observed between survival rate and *CDKN2A/B* deletion, *EGFR* gene alteration, *TP53* mutation, or gain of chromosome 7 and loss of chromosome 10 (*p* > 0.05) (Figure 5, Table 5 and Table S3).

In multivariate analysis, only precedent history of other cancer remained a statistically significant predictor (HR = 4.01, 95%CI 1.85–8.7, *p* = 0.0004) (Table 5).

### 2.6. Molecular-Matched Targeted Treatment

The results of genomic profiling were applied for the personalization of treatment. Actionable molecular targets were identified and used for targeted therapy in 9 patients with glioma and glioblastoma. The patients’ detailed characteristics are presented in Table 6. The candidates for targeted therapy (9/131 or 7% of the total group) were selected according to the recommendations provided by FoundationOne genetic testing. The targeted therapy was prescribed by a medical consilium as off-label treatment (compassionate use) after progression or relapse, starting when other treatment options had been exhausted.

**Figure 6:**
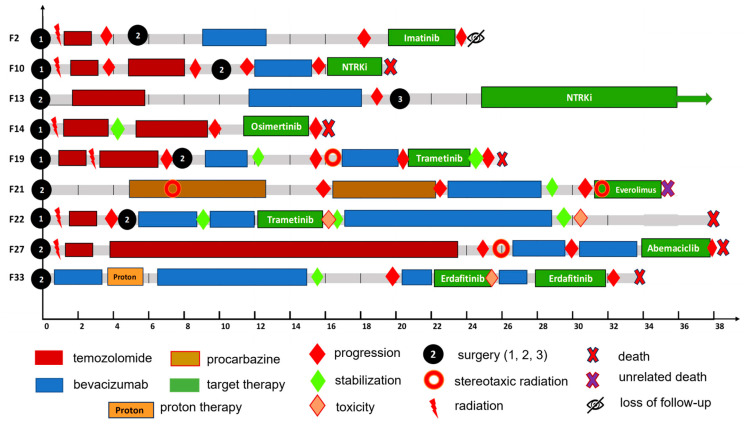
Follow-up of patients during treatment with targeted therapies.

Treatment follow-up data are presented in Figure 6.

Three patients were diagnosed with *IDH*—wildtype (Grade 2) or *IDH*—mutant diffuse astrocytoma (Grade 3 and 4). Other six patients had *IDH*—wildtype glioblastoma. All patients received standard-of-care radiation therapy to a total dose of 60 Gy with concomitant temozolomide. The mean time between initial surgical resection and the start of adjuvant therapy was 1.1 months (range: 0.6–2.0 months). Several patients (F10, F14, F27, F33) received six additional cycles of temozolomide according to the Stupp protocol, and F27 received 24 cycles of temozolomide overall. All patients except F14 received bevacizumab in mono- or combined therapy with chemotherapeutic (temozolomide, irinotecan, paclitaxel, carboplatin, lomustine) or immunotherapeutic (nivolumab) drugs before or after molecular targeted therapy. A patient with giant-cell glioblastoma benefited from procarbazine for a long period of time, then received combined therapy with bevacizumab and nivolumab, followed by targeted therapy with everolimus, due to the presence of *NF1* mutation. Most patients underwent two or three surgical resections of the tumor; also, stereotaxic radiotherapy was applied. The mean time from first surgery to start of targeted treatment was 27.4 months (range: 11.9–36.8 months), and the mean duration of treatment was 6.03 months (range: 1–30 months). One patient (F13) receiving entrectinib remained alive with progression-free survival for 30 months and OS for 66 months. Seven patients died, and one was lost of follow-up; the average survival time was 34.7 months (range: 15.4–78.7 months). One death was due to COVID-19 infection (F21), other deaths resulted from disease progression. In two cases (F22 and F33), severe toxicity developed during drug administration, which led to interruptions or cancellation of the targeted drugs (trametinib and erdafitinib).

## 3. Discussion

### 3.1. Mutational Profiles of Diffuse Glioma Samples

In our study, we performed a comprehensive investigation of mutations and chromosomal aberrations in 131 samples of glioma patients and compared the molecular findings with clinical characteristics of the patients. Patients with low-grade glioma (LGG) represented 14% of the total cohort and were significantly younger than HGG patients; the median age of LGG patients was 36.5 years, compared with 59 years in HGG patients. Overall survival in LGG patients was significantly better than in HGG patients. At the moment of last observation, 83% of our LGG patients were alive. Among LGG patients, *IDH*—mutant gliomas occurred in 78% cases, consistent with other studies. As previously shown, *IDH*—mutant gliomas comprise approximately 70–80% of histologically low-grade gliomas, commonly occuring in patients under the age of 50, and generally responding better to treatment than *IDH*—wildtype gliomas [38,39,40,41,42].

Oligodendrogliomas (ODGs) are defined as gliomas with a mutation in *IDH1/2* genes and an unbalanced translocation between chromosomes 1 and 19 (1p/19q-codeletion); they are graded on a scale of 2–3, based upon histological features such as mitotic activity and presence of microvascular proliferation or necrosis [27]. In our study, *IDH1* mutation R132H and 1p/19q codeletion were found in 71% of patients diagnosed with ODG, while one patient carried no *IDH* mutation and only 1p deletion. In two cases, mutations in the *CIC* gene were revealed, which is a highly specific marker of ODG and may occur in up to 50% of cases [43].

*IDH*—mutant low-grade gliomas without 1p/19q codeletion are defined as astrocytoma and graded on a scale of 2 to 3 [27]. We found *IDH* mutations in 72% of patients; most cases were *IDH1* R132H, and one case was *IDH2* R172S. *IDH2* mutations are substantially rare events in gliomas, accounting for 0.3–5.2% of cases [44]. Other molecular alterations included mutations in the *TP53*, *ATRX*, *PIK3CA*, *PIK3CB*, *PTEN* genes, and *CDKN2A* deletion, which have been found to be specific to low-grade gliomas [27,42]. *TERT* promoter mutations were found in 28% of LGG patients. In one tumor with wildtype *IDH*, a *TRIM33-NTRK1* translocation was identified in a young patient aged 18 years old. Neurotrophic tropomyosin receptor kinase (NTRK) family gene fusions are rare, found in only 1% of gliomas across adult and pediatric patients, and these LGGs exclusively harbored *NTRK* fusions without additional genetic alterations [45]. Three other cases of *IDH*—wildtype LGG were diagnosed as diffuse astrocytoma according to obvious morphologic criteria. One of these cases had a *TERTp* mutation and could be reclassified as a Grade 4 glioblastoma according to the 2021 WHO CNS. All these patients had a median survival time of 73 months, and the patient with *TERTp* mutation died after 41 months of treatment. Some authors consider such cases within the 2021 WHO CNS classification as a separate subtype of *IDH*—wildtype Grade 2–3 diffuse astrocytoma [29]. Surgical resection represents a critical first step in the treatment of *IDH*—mutant gliomas, and more extensive resections are associated with improved OS [46]. Among our LGG patients, two resections were made for 61% of patients, and the mean time between first and second resection was 67 months (ranging from 7 to 117 months).

In the HGG group, *IDH1* R132H mutation was found in three patients with Grade 4 diffuse astrocytoma. Mutational spectra were specific for diffuse astrocytoma and included mutations in the *TP53* and *ATRX* genes, one *TERTp* mutation, and one *CDKN2A/B* deletion, which is considered a hallmark of Grade 4 astrocytoma [27]. All patients were younger than 30 years of age and had undergone 2 or 3 resections. Genetic testing was performed for recent recurrent tumors, and the primary tumors of these patients were defined as diffuse grade 2 or 3 astrocytoma after histological examination. It was reported that development of a high-grade glioma phenotype was associated with temozolomide-driven hypermutation and acquired alterations of the cell cycle regulators CDKN2A and CCND2, which have been shown to occur exclusively in post-radiation *IDH*—mutant gliomas [47,48].

*IDH*—wildtype glioblastoma remains the most common malignant adult-type diffuse glioma [27,49]. Mutational profiling of glioblastoma samples from our patients included recurrent mutations *TERTp* (71.8%), *PTEN* (37.3%), *TP53* (27.3%), *EGFR* (13.6%), *NF1* (11.8%), *RB1* (11.8%), *PIK3CA* (4.5%), *PIK3R1* (4.5%), *PTPN11* (4.5%), *SPTA1* (3.6%), *PIK3CB* (1.8%), and *MTOR* (1.8%). Also, deletion of *CDKN2A/B* (32.7%), deletion of *PTEN* locus (37.3%), amplification of the *EGFR* locus (38.2%), amplification of chromosomal regions 4q12 (10%), 12q13 (8.1%), and 12q15 (6.3%), deletion of 17p13 (15.4%), gain of chromosome 7 (22%), and loss of chromosome 10 (46%) were common in glioblastoma specimens. In chromosomal region 4q12, *PDGFRA*, *KIT*, and *KDR* genes are localized, which play critical roles in development of glioblastoma [50,51]. Amplification of chromosomal regions 12q13 and 12q15 involves *CDK4* and *MDM2* genes, respectively. The *CDK4* gene has been suggested to regulate cell invasion and stemness in glioblastoma [52], while the *MDM2* gene is an important player in the p53 pathway [53]. Loss of heterozygosity in chromosomal region 17p13, where the *TP53* gene is localized, occurs in many cancers and is usually associated with unfavorable prognosis [54]. The mutation frequencies identified in our cohort of glioblastoma patients are consistent with the results of other studies [16,38,55]. Interestingly, different patterns of mutation have been described in some Asian glioblastoma patients [56,57].

### 3.2. Signaling Pathways Affected in Glioma Samples

The most frequently altered genes in gliomas are involved in different signaling pathways associated with cancer development and progression [58]. Overexpression of tyrosine kinase receptors (RTKs), namely EGFR, PDGFR, VEGFR, and FGFR, and their ligands is among the most frequent alterations in glioma tumorigenesis, leading to aberrant activation of their downstream molecular pathways [18,59]. All of these signaling pathways form a complex network that regulates numerous cellular processes. In our sample, amplification or mutation of the *EGFR* gene, as well as amplification of the *PDGFRA* and *KIT* genes, potentially leading to their overexpression, were prevalent in HGG *IDH*—wildtype glioblastoma. Also, mutations and rearrangements affecting *PTEN*, *PI3K* family, and *MTOR* genes, the key participants in the PI3K/AKT/mTOR signaling axis, were more frequent in *IDH*—wildtype glioblastoma (80%) compared with LGG (38%). In another study, genomic analysis also predicted PI3K/AKT/mTOR pathway activity in 21.7% of *IDH*—mutant diffuse glioma. However, when protein expression was examined, activation of the PI3K/AKT/mTOR signaling pathway was detected in 56.6% of samples [60,61]. Thus, integrated studies allow a more adequate estimation of the levels of signaling pathway activity in tumor cells.

The RAS/MAPK signaling pathway also plays an important role in gliomagenesis, and the *NF1* gene, which is a negative regulator of the RAS oncogene, is most commonly affected in adult-type gliomas [16,59,62]. In our study, loss-of-function mutations in the *NF1* gene were found both in LGG (6%) and in HGG (12%) samples. The *TP53* gene is one of the most frequently deregulated genes in cancer [17], particularly in gliomas, and protein 53 is a central part in the TP53/MDM2/MDM4 pathway, which becomes active in response to DNA damage or other cellular changes. Clinically relevant mutations in the *TP53* gene were more common in LGG (44%) compared with HGG (27%) samples, probably because impairment of the TP53/MDM2/MDM4 pathway is more characteristic of LGG.

The protein product of the *CDKN2A* gene, p16, plays a critical role in cell cycle regulation via its interaction with the retinoblastoma tumor suppressor encoded by the *RB1* gene [63]. Also, cyclin-dependent kinase 2 types A and B (CDKN2A/B) are involved in both the p16^INK4a^/CDK4/RB1 pathway and the TP53/MDM2/MDM4 pathway [20,59,62]. In our study, *CDKN2A/B* deletion or *RB1* mutation, which may affect the p16^INK4a^/CDK4/RB1 pathway, were found predominantly in HGG (44%) compared with LGG samples (5%).

Thus, in the entire HGG sample, and in *IDH*—wildtype glioblastoma, representing 97% of cases, genomic profiling predicted very high levels of impairment in the most important cellular signaling pathways.

### 3.3. Mutational Profiles of Primary and Recurrent Glioblastoma

We compared mutational profiles of primary glioblastoma tumors and recurrent glioblastoma obtained after second or third resection. We did not find substantial differences in mutation spectra and frequency between primary and recurrent tumors for most studied genes, except *TERT* and *EGFR*. In our study, *TERTp* mutations (77% vs. 56.4%, *p* = 0.01) and *EGFR* amplification (47.9 vs. 20.5%, *p* = 0.01) were more frequent in primary glioblastoma in comparison with recurrent tumor samples. Previously, it was shown that *PTEN* alterations prevailed in primary glioblastoma, with *EGFR* amplification, by contrast, in recurrent tumors [38]. In other work, it was shown that copy number variation (CNV) was more frequent in recurrent tumors and *CDKN2A/B* loss was significantly enriched in the sample [64], but that study included *IDH1/2* mutant glioma, where *CDKN2A/B* deletion is a marker of tumor progression. Glioblastoma is a tumor with a low mutational load, so the evolution of the tumor and the emergence of new mutations is much slower than in other cancers. In our study, the mean time between first and second resections was 16 months (ranging from 3 to 89 months). Meanwhile, it is suggested that molecular testing should be performed from the most recent tumor tissue sample whenever possible, to provide the most accurate molecular analysis [65]. Previously, increased mean TMB in recurrent samples was found to be due to hypermutagenesis induced by temozolomide treatment [66]. In our study, TMB varied from 0 to 18 mut/Mb, with a median value of 3 mut/Mb, but one primary glioblastoma sample with *MSH6* mutations had TMB equal to 82 mut/Mb. A hypermutator phenotype has previously been described in primary and recurrent glioblastoma samples with mutations in at least one of the mismatch-repair (MMR) genes (*MLH1*, *MSH2*, *MSH6*, and *PMS2*) [22,67]. Higher mutational burden was reported to be associated with response to nivolumab or pembrolizumab in other cancers [68], but only small subsets of glioma patients with MMR deficiency and high TMB were likely to benefit from monotherapy with immune checkpoint inhibitors [69].

### 3.4. Clinical Characteristics and Overall Survival

In our cohort of 131 glioma patients, 13 individuals had another cancer which was precedent to the CNS tumor. In all cases, the CNS tumor was *IDH*—wildtype glioblastoma. According to Surveillance, Epidemiology, and End Results (SEER) program data, primary malignant brain tumors following systemic malignancies developed in 0.09% of patients, and glioblastoma was the most common pathological type, accounting for 68.4% of all second primary CNS tumors [70]. More often, second primary CNS tumors developed in patients with prostate cancer, breast cancer, melanoma of the skin, or urinary bladder cancer. In our study, the most frequent first malignancies were colorectal cancer, breast cancer, and bladder cancer. The most common genetic alterations in SPGB patients were *CDKN2A/B* deletion (46%), *TERTp* mutation (61%), *PTEN* deletion (54%), *TP53* mutation (23%), and *EGFR* amplification (54%).

Survival analysis showed that in the whole cohort of glioma patients, low tumor grade and *IDH1/2* mutation were independent factors of better prognosis. Thus, our patients with *IDH*—wildtype glioblastoma had the worst prognosis compared with LGG *IDH*—wildtype patients and HGG *IDH*—mutant patients, in agreement with many other studies [35,36,71,72]. We found that having a history of cancer with another localization preceding glioblastoma dramatically reduced survival, and this poor prognostic value persisted irrespective of other features.

Treatment of glioblastoma is based primarily on the maximum possible resection; the prognosis is considered favorable when at least 80% of the tumor volume is removed [73]. Nevertheless, gross total resection (GTR) is not always achievable, because it may affect functionally significant and sensitive areas of the brain. In these cases, the use of preoperative and intraoperative neuroimaging techniques is of particular importance [74]. Functional magnetic resonance imaging (fMRI) has been widely used in neurophysiologic studies and has proven particularly useful in preoperative neurosurgical planning for patients previously unresectable due to uncertain risk of neurologic deficit [74,75].

Second surgery is a treatment choice for a limited number of patients, usually due to poor clinical status or involvement of critical areas of the brain, and the rate of patient candidates for a second surgery is approximately 20–30% [76]. Current data on the role of subsequent surgical interventions in recurrent glioblastoma are still lacking clinical evidence [77]. At the same time, several studies have demonstrated the potential benefits of reoperation in the treatment of recurrent glioblastoma, resulting in increased survival [78,79]. In our study, the number of surgical interventions mattered in patients with glioblastoma, and prognosis was better in those patients who underwent more than one resection. This may be associated with the better initial physical condition of the patients and slower tumor progression, because it is believed that patients undergoing re-operation are usually younger, with better Karnofsky performance scores (KPS ≥ 70) and smaller tumor size [80]. In addition, increased survival of patients with recurrent glioblastoma may be associated with the combination of re-operation, re-irradiation, and chemotherapy as treatment modalities, resulting in a more pronounced effect compared with the use of these treatment options alone [79].

Since there is no well-established second-line chemotherapy scheme, patients are usually treated under investigational regimens. Bevacizumab, which binds with high specificity to VEGF and blocks angiogenesis, has demonstrated a high response rate in the treatment of disease progression [81]. However, it has been observed that this effect does not last long and may be due to changes in vascular permeability. Bevacizumab has shown improved progression-free survival in several clinical studies, but OS benefits have not been consistently observed [82]. In our study, a marked increase in the survival probability of patients treated with bevacizumab was observed during the first 2 years, but this difference almost disappeared after approximately 3 years. To maximize benefits from bevacizumab treatment, combinations of bevacizumab with other treatment options are used to enhance synergistic anti-tumor effects [83]. In our study, combined bevacizumab therapy was applied to 34% of patients. Another critical parameter considered to influence the efficacy of combined radiation therapy and temozolomide is the methylation status of the *MGMT* promoter, which has been shown to be the only molecular feature having both prognostic and predictive values in glioblastoma patients [84]. Patients with unmethylated *MGMT* have been shown to receive modest benefit from chemotherapy, and their response to radiation therapy may also be reduced [85,86]. In our study, median survival for patients with methylated *MGMT* was longer, but not significantly so, probably due to limited number of participants with known methylation status.

### 3.5. Impact of Mutations to Overall Survival

Several studies in the past decade have investigated the association of patient survival with mutations in the genes *TP53*, *PTEN*, *EGFR*, and *TERT* promoter, and *CDKN2A/B* deletion [87,88,89]. The role of homozygous *CDKN2A/B* deletion as an independently prognostic biomarker for worse survival among *IDH*-wildtype glioblastoma has been indicated [87,88], but the data concerning other genes are very controversial. We found a significant association of patient survival with *TERTp* mutation and *PTEN* deletion, both of which were biomarkers of worse prognosis; while other genetic alterations did not significantly affect clinical outcomes. Also, *TERTp* mutations were rarer in our patients with recurrent glioblastoma, with better prognostic value. Several studies have also reported a negative independent prognostic impact of *TERTp* mutations [24,90,91], while others have suggested that the adverse impact of *TERTp* mutation correlates with the presence of associated molecular and clinical factors such as advanced age, wildtype *IDH* status, and unmethylated *MGMT* promoter [92,93]. The same situation has been observed for *PTEN* alterations. In several studies, *PTEN* mutation was associated with improved overall survival [38,88], whereas other reports demonstrated negative impacts of mutated *PTEN* on clinical outcomes in *IDH*—wildtype glioblastoma patients [94,95]. The 10q23/*PTEN* homozygous deletion was found to be associated with shorter survival, particularly in patients older 45 years [96]. At the same time, the impact of mutations in the *CDKN2A*, *CDKN2B*, *EGFR*, *PTEN*, *TERT* promoter, and *TP53* genes has been shown to correlate with the volume of surgery, and the combination of mutations in any of these genes with GTR may result in improved survival [88].

Thus, among genetic factors of prognosis, *IDH1/2* mutations had the strongest impact. Genetic changes in other frequently mutated genes had a much more modest influence on the overall survival of glioma patients. At the same time, understanding the molecular nature of the disease and identifying specific damaged genes offers great potential for targeting intracellular processes. The number of potentially actionable molecular alterations revealed in glioma patients may be rather high (up to 76% of cases) [39], but in reality, only about 10% of patients are considered to benefit from molecular targeted drugs [38]. In fact, the most frequent molecular alterations identified in glioma and glioblastoma patients (*CDKN2A/2B*, *PTEN*, *TP53*, *TERT*, *PIK3CA/B* and *EGFR* alterations) have no clinically active drugs for treatment of CNS tumors.

### 3.6. Molecular-Targeted Therapy

Among our patients treated with targeted drugs, the best results were shown with the NTRK inhibitor entrectinib, where a durable complete response was achieved. Entrectinib has previously been tested in advanced and metastatic solid tumors in adults [97] and in pediatric patients with CNS tumors harboring *NTRK* aberrations [98]. Our patient receiving entrectinib also had an initially favorable clinical background (young age and low-grade glioma); in another patient with glioblastoma in whom increased NTRK expression was detected by immunohistochemistry, the use of the NTRK inhibitor larotrectinib had no beneficial effect. In other studies, promising results have been obtained with the pan-FGFR kinase inhibitor erdafitinib [99]. In our study, a patient treated with erdafitinib developed toxicity that led to treatment interruptions. Trametinib is a MEK1/2 inhibitor that has been found to be effective for treatment of *BRAF* V600E-mutated gliomas [100], and it is also being tested in the treatment of patients with *NF1* mutations [101]. Our study did not show a positive effect of trametinib in the treatment of glioblastoma patients. In another patient with giant-cell glioblastoma and *NF1* mutation, the use of everolimus (mTOR inhibitor) may have had an impact, but the patient died due to COVID-19 infection. Since a patient may carry several actionable mutations, including those affecting tumor progression, the complex interactions of these may influence the outcome of treatment with a single targeted drug. Furthermore, glioma may be characterized by significant inter- and intratumor genomic heterogeneity and during progression, recurrent tumors may switch to a different phenotype associated with changes in cellular, histological, and molecular features [47,102].

### 3.7. Limitations of the Study

This study has several limitations. First, we used two different target panels when sequencing the samples, which differed in the number of genes examined. Nevertheless, both panels were highly representative and included almost all currently known clinically relevant genes associated with the development of CNS tumors.

Second, diffuse gliomas and especially glioblastomas are characterized by a high degree of intratumoral heterogeneity; so, different tumor sites may differ in their mutational profile. To minimize such errors, we performed repeated studies of the mutational profile for some samples.

Third, in this study, we did not consider the extent of intraoperative interventions, despite the fact that the extent of resection may influence clinical outcomes. This was partly due to the study design, as we included patients who underwent their first surgery at other centers, and these data were not available.

Fourth, our sample size was not very large, and this may not have allowed statistically significant differences to be achieved when analyzing the influence of various clinical and genetic factors on survival. The small sample size was due to the fact that most of the samples were prospective, as we took not only a tumor sample but also a peripheral blood sample as normal tissue. This allowed us to more accurately identify somatic mutations and chromosomal rearrangements in tumor samples.

## 4. Materials and Methods

### 4.1. Patients

We analyzed patients with new histopathological diagnoses of glioma according to the 2021 WHO criteria, in N.N. Blokhin Russian Cancer Research Center of the Ministry of Health of the Russian Federation, over a period between January 2019 and August 2024. Tumor histological material obtained before 2021 was revised to clarify the diagnosis. The follow-up period ended on 31 August 2024. The study protocol was approved by the institutional ethics committee. All patients with recurrent disease were discussed by the multidisciplinary tumor board. Medical records were evaluated in terms of age, sex, comorbidities, clinical symptoms, tumor location, surgical intervention, treatment protocol, tumor histology, and laboratory tests. Fresh tumor samples were received during surgery, divided into pieces, frozen, and stored until use at −70 °C. To prepare formalin-fixed paraffin-embedded (FFPE) samples, tumor tissue was fixed with 4% formaldehyde solution and embedded in paraffin blocks according to a standard method for preparing histologic specimens. Peripheral venous blood was used as a normal tissue sample.

### 4.2. Next-Generation Sequencing

Genomic DNA was isolated from fresh-frozen tissue and leukocytes using the QIAamp DNA Mini Kit (Qiagen, Hilden, Germany), and DNA was extracted from paraffin sections of tumor tissue fixed by formaldehyde using a QIAamp DNA FFPE Tissue Kit (Qiagen, Hilden, Germany) according to the manufacturer’s protocols. To establish the multigene panel, we selected 812 genes known to be involved in cancer development and progression. The panel was designed using the HyperDesign online service provided by Roche (Roche, Basel, Switzerland), which included exons of selected genes and adjacent intronic regions containing splicing sites as well as 5′-UTR regions. Libraries were prepared using the KAPA HyperPrep Kit (Roche, Basel, Switzerland) following a standard protocol. For targeted sequence selection, hybridization with a custom panel was performed according to the manufacturer’s protocol (Roche, Basel, Switzerland). Paired-end sequencing was performed on the NextSeq2000 platform from Illumina (Illumina, San Diego, CA, USA) with an average coverage of 400–500×. For 33 patients, FoundationOne^®^CDx testing was performed, which covered a subset of 324 essential tumor-associated genes [103] (Foundation Medicine GmbH, Penzberg, Germany).

### 4.3. Bioinformatics and Statistical Analysis

Paired-end reads were aligned to the reference genome (hg37) using the BWA-MEM2 algorithm. Duplicate sequences were then identified and removed using the Picard MarkDuplicates program. Then, recalibration of the quality scores of the bases was performed and identification of genetic variants was performed using Genome Analysis Toolkit (GATK) tools: BQSR for recalibration of scores and HaplotypeCaller for variant calling. To identify somatic variants, tumor samples were compared with matched blood samples using Mutect2 somatic variant caller. Interpretation of all identified variants was performed using open access databases: ClinVar, Varsome, and Franklin by Genoox, utilizing the American College of Medical Genetics and Genomics (ACMG) interpretation standards and guidelines. A variant was considered pathogenic or likely to be pathogenic if it was noted in at least one database. Comparative analysis of β-allele frequencies (BAF) in paired normal–tumor samples was used to estimate copy number variations (CNVs) in different regions of the genome. All germline heterozygous variants with variant allele frequency (VAF) between 40% and 60% in the normal sample and coverage of at least 30 reads in both normal and tumor tissue were selected. Cases of probable copy number variation were defined if tumor VAF values were >40–60% and differences in VAF between normal and tumor tissue were >25% (*p* < 0.05). BAF analysis allows the detection of copy number changes but does not always make it possible to determine the character (deletion or amplification); thus, additional analysis of coverage depth was performed (Figure S1).

### 4.4. Statistics

Overall survival (OS), which was assessed as the time between the first surgery and death from any cause, was studied as an outcome. Statistical analysis and visualization of survival data were performed using R version 4.3.3 (R Foundation for Statistical Computing, Vienna, Austria), using the packages “survival”, “survminer”, and “ggplot2”. The Kaplan–Meier method was used to construct survival curves and determine median OS (with 95% CI) and 1-, 2-, and 5-year OS rates. Survival was compared between groups using the log-rank test. The Cox proportional hazards model was used to determine HR (with 95% CI), first performing univariate analysis, and then, predictors with a *p*-value < 0.05 were used in multivariate analysis. To calculate odds ratio (OR), Fisher’s two-sided criterion was used.

## 5. Conclusions

Our study demonstrates the clinical utility of next-generation sequencing in patients with glioma. The efficacy of therapy and clinical outcomes are primarily dependent on the degree of tumor malignancy and the *IDH* mutational status. It has been shown that a lower degree of tumor malignancy and the presence of *IDH1/2* mutations are independent factors of better prognosis, which is consistent with the results of other studies. In patients with *IDH*—wildtype glioblastoma, a previous history of cancer at another localization was of great importance, and dramatically decreased overall survival. In addition, treatment details (one or more surgeries, bevacizumab therapy) were also prognostic factors. Patients with recurrent glioblastoma who underwent surgery more than once had higher overall survival compared with patients with primary glioblastoma and only one resection. Bevacizumab therapy improved survival rates during the first 2 years, but this effect disappeared over a longer period of time. The presence of *TERTp* mutations and *PTEN* deletion decreased overall survival in glioblastoma patients, while the influence of other genetic markers was not significant. The results of genomic profiling were used to select molecular targeted therapies for a limited cohort of patients. The most successful results were achieved with entrectinib in a patient with *NTRK* fusion, where overall survival exceeded 66 months with a treatment duration more than 30 months. Therefore, NGS analysis may be in demand, involving more patients in clinical trials of new targeting agents in order to find effective approaches for treating groups of patients with poor survival rates.

## Figures and Tables

**Table 1:** Clinical characteristics of glioma patients involved in the study.

Characteristics		Total, n (%)
Gender		131 (100%)
	Male	69 (53%)
	Female	62 (47%)
Median age (range), years		58 (from 18 to 78)
Distinct glioma grades		
Grade 2–3 (LGG)		18 (14%)
Median age (range), years		36.5 (18–64)
Grade 4 (HGG)		113 (86%)
Median age (range), years		59 (22–78)
Tumor distribution by lobe		
Multilobar (two or more lobes)		48 (36.5%)
	Frontal	36 (27%)
	Temporal	37 (28%)
	Parietal	8 (6%)
	Occipital	2 (1.5%)
Surgery		
First surgery (primary glioma)		80 (61%)
Surgery at relapse (recurrent glioma)		51 (39%)
Personal history of other cancer		
Yes		13 (10%)
No		118(90%)
Radiotherapy		
	With TMZ	122 (93%)
	Without TMZ	3 (2.4%)
TMZ monotherapy		6 (4.6%)
Bevacizumab (BEV)		
	Monotherapy	31 (24%)
Combined therapy		44 (34%)
	Not received	56 (42%)
Outcome		
	Dead	81 (62%)
	Alive	50 (38%)

**Table 2:** Frequency of molecular alterations in primary and recurrent *IDH*—wildtype glioblastoma samples (Rec: recurrent; Prim: primary). Significant difference is marked by (*).

Gene Alteration	All (n = 110)	%	Primary (n = 71)	%	Recurrent (n = 39)	%	OR (95%CI) (Rec. vs. Prim.)	*p*-Value
*CDKN2A/B* deletion	36	32.7	28	39.4	8	20.5	0.39 (0.15–0.98)	0.06
*TERT*	79	71.8	57	77.0	22	56.4	0.32 (0.13–0.75)	0.01 *
*PTEN* mutation	41	37.3	25	35.2	16	41.0	1.28 (0.57–2.86)	0.68
*PTEN* deletion	47	42.7	35	49.3	12	30.7	0.52 (0.23–1.19)	0.22
*TP53*	30	27.3	16	22.5	14	35.8	2.1 (60.91–5.15)	0.11
*EGFR* mutation	15	13.6	13	18.3	2	5.1	0.24 (0.05–1.31)	0.07
*EGFR* amplification	42	38.2	34	47.9	8	20.5	0.28 (0.11–0.69)	0.01 *
*EGFR* rearrangement	57	52.0	48	68.0	10	25.6	0.38 (0.22–0.66)	0.0001 *
*NF1*	13	11.8	7	9.9	6	15.4	1.27 (0.38–4.19)	0.75
*RB1*	13	11.8	6	8.5	7	17.9	2.74 (0.85–8.85)	0.12
*PIK3CA*	5	4.5	3	4.2	2	5.1	1.39 (0.22–8.73)	0.66
*PTPN11*	5	4.5	4	5.6	1	2.5	0.50 (0.05–4.64)	1.00
*ATRX*	4	3.6	3	4.2	1	2.5	0.68 (0.07–6.74)	1.00
*PIK3R1*	5	4.5	3	4.2	2	5.1	1.39 (0.22–8.72)	0.66
*SPTA1*	4	3.6	3	4.2	1	2.5	0.68 (0.07–6.74)	1.00
4q12 amplification	11	10.0	9	12.6	2	5.1	0.18 (0.02–1.49)	0.98
12q13 amplification	10	8.1	7	9.8	3	7.7	0.87 (0.21–3.58)	1.00
12q15 amplification	7	6.3	5	7.0	2	5.1	0.81 (0.14–4.40)	1.00
17p13 deletion	17	15.4	11	15.4	6	15.4	1.15 (0.38–3.39)	0.78
Chromosome 7 gain	22	20.0	15	21.1	7	17.9	0.94 (0.35–2.59)	1.00
Chromosome 10 loss	46	41.8	34	47.8	12	30.7	0.58 (0.26–1.35)	0.23

**Table 3:** Characteristics of patients with SPGB (SPGB: second primary glioblastoma; GB: glioblastoma; T1: time between first tumor and GB in years; T2: time between first GB and second GB in months).

ID	1st Tumor (age)	2nd Tumor (Age)	T1 (1st Tumor—GB1) (y)	T2 (GB1—GB2) (m)	Somatic Mutations in GB	Outcome	Survival after GB1 (Month)
G10	Melanoma (47)	GB (65)	18	-	*PTEN*, *EGFR amp*, *PTEN del*	Dead	4
G11	Breast cancer (50)	GB (59)	9	-	*TP53*, *PTEN del*, *EGFR amp*, *CDKN2A/B del*	Dead	9
G18	Colorectal cancer (62)	GB (62)	0	-	*TERTp*, *TP53*, *RB1*	Dead	4
G63	Breast cancer (64)	GB (66)	2	-	*TERTp*, *PTEN del*, *PTPN11*, *BCOR*	Dead	5
G65	Colorectal cancer (56)	GB (61)	5	-	*EGFR*	Alive	20
G67	Colorectal cancer (55)	GB (57)	2	-	*TERTp*, *PTEN del*, *EGFR amp*	Dead	2
G69	Myeloma (64)	GB (66)	2	-	*TERTp*, *PTEN*, *EGFR amp*, *PTEN del*, *CDKN2A/B del*	Dead	10
G70	Bladder cancer (64)	GB (76)	12	-	*TERTp*, *EGFR amp*, *PTEN del*	Dead	6
G75	Colorectal cancer (56)	GB (58)	2	-	*TERTp*, *EGFR*, *PIK3CB*	Alive	4
G84	Bladder cancer (69), prostate cancer (69)	GB (69)	4 months	-	*TP53*, *ATRX*, *CDKN2A/B del*	Alive	6
G86	Renal cancer (70)	GB1 (70), GB2 (72)	0	27	*ATM*	Alive	33
F30	Breast cancer (63)	GB1 (72), GB2 (72)	11	8	*TERTp*, *PIK3CA*, *NRAS*, *FGFR1*	Dead	19
F32	Thyroid cancer (51)	GB (55)	4	-	*TERTp*, *EGFR mut*	Dead	10

**Table 4:** Univariate and multivariate Cox regression analysis of OS in glioma patients depending on tumor grade and *IDH* status.

	Univariate		Multivariate	
Variable	HR (95%CI)	*p*-Value	HR (95%CI)	*p*-Value
Age (18–78) years	1.042 (1.023–1.062)	<0.001		
Grade (LGG vs. HGG)	0.085 (0.026–0.277)	<0.001	0.1683 (0.047–0.608)	0.0065
IDH1/2 (mut vs. wt)	0.099 (0.031–0.32)	<0.001	0.2489 (0.069–0.89)	0.03263

**Table 5:** Univariate and multivariate Cox regression analysis of overall survival in patients with glioblastoma (mut: mutation; del: deletion; amp: amplification). Significant difference is marked by (*).

	Univariate			Multivariate		
Variable	HR	95% CI	*p*-Value	HR	95% CI	*p*-Value
Sex (male)	1.16	0.73–1.83	0.51			
Precedent history of other cancer	2.70	1.32–5.52	0.006*	4.01	1.85–8.7	0.0004 *
Recurrent surgery	0.53	0.32–0.85	0.009*	0.70	0.41–1.19	0.19
Unilobar glioblastoma	0.87	0.54–1.42	0.59			
Bevacizumab therapy	0.62	0.39–0.98	0.04*	0.75	0.46–1.21	0.24
Methylated MGMT	0.70	0.42–1.17	0.18			
*CDKN2A/B* deletion	1.04	0.64–1.69	0.87			
*TERTp* mutation	1.98	1.13–3.47	0.02*	1.8	0.95–3.38	0.069
*PTEN* deletion	1.66	1.05–2.64	0.03*			
*PTEN* mutation	1.15	0.73–1.82	0.54			
*PTEN* alteration (mut or del)	1.70	1.05–2.74	0.03*	1.5	0.88–2.54	0.14
*TP53* mutation	1.25	0.75–2.06	0.39			
*EGFR* alteration (mut or amp)	1.38	0.87–2.17	0.17			
Chr 7 gain	1.67	0.77–3.61	0.20			
Chr 10 loss	1.35	0.77–2.36	0.29			

**Table 6:** Description of patients undergone molecular-targeted therapy (GB: glioblastoma; Surg: surgery; IHC: immunohistochemistry; LFU: loss of follow-up; mut: mutation; amp: amplification).

ID	Age, Sex	Diagnosis	Surg (n)	Molecular Target	Drug	T1 (m)	T2 (m)	Dur (m)	O	S (m)
F2	23, f	GB IDHwt	2	*PDGFRA* amp	Imatinib	0.8	20	4	LFU	24
F10	62, m	GB IDHwt	2	*PTEN*, *NTRK* (expression IHC)	Larotrectinib	2	15.6	2	Dead	19.3
F13	18, f	DA G2 IDHwt	3	*TRIM33–NTRK2*	Entrectinib	0.6	36	30	Alive	66
F14	55, f	GB IDHwt	1	*EGFR* (mut, amp)	Osimertinib	1.1	11.9	3.3	Dead	15.4
F19	34, f	GB IDHwt	2	*TP53*, *NF1* (sub)	Trametinib	1	21.7	3	Dead	25.8
F21	52, f	GB IDHwt (giant cell)	2	*NF1*, *TP53*, *RB1*	Everolimus	1	75.4	3	Dead	78.7
F22	65, f	GB IDHwt	2	*NF1*, *TERTp*, *CDKN2A/B del*	Trametinib	1.4	13.4	1	Dead	38.5
F27	32, f	DA G4 IDHmut	2	*IDH1*, *CDK6* amp, TP53, CDKN2A del	Abemaciclib	1	36.8	1.5	Dead	38.5
F33	65, f	DA G3 IDHmut	2	*FGFR3-PDE4DIP* *TERTp*	Erdafitinib	1	22.3	6.5	Dead	33.8

## Data Availability

Data are available on request.

## Supplementary Materials

The following supporting information can be downloaded at https://www.mdpi.com/article/10.3390/ijms252313004/s1.
