# Supplementary material for: Genomic Profiling in Glioma Patients to Explore Clinically Relevant Markers
Source: Int J Mol Sci. 2024 Dec 3;25(23):13004. doi: 10.3390/ijms252313004 (PMC11641329; doi:10.3390/ijms252313004)
Supplement: Supplementary file 1 [file ijms-25-13004-s001.zip › Table S2_Frequency of genetic alterations in LGG and HGG groups.pdf]

Table S2. Frequency of genetic alterations in LGG and HGG samples compared with Glioblastoma multiforme TCGA, PanCancer Atlas (https://www.cbioportal.org)

**Samples**	**LGG (n=18)**	**HGG (n=113)**	**TCGA, 2018 (n = 592)**
CDKN2A	6%	33%	56%
IDH1/2	78%	3%	6%
TERT	28%	71%	77%
PTEN_mut	11%	36%	33%
PTEN_del	6%	42%	10%
PTEN_all_rearr	11%	60%	NA
TP53	44%	27%	31%
EGFR_mut	0%	13%	24%
EGFR_amp	0%	37%	44%
EGFR_all_rearr	0%	40%	NA
NF1	6%	12%	12%
RB1	0%	12%	10%
PIK3CA	17%	4%	10%
PTPN11	0%	4%	3%
ATRX	33%	4%	10%
PIK3R1	6%	4%	10%
SPTA1	0%	4%	10%
PIK3CB	6%	2%	3%
CIC	11%	0%	2%
MTOR	0%	2%	2%
Chr 10 del	6%	40%	NA
Chr 9 del	6%	18%	NA
Chr 1 del	33%	11%	NA
Chr 17 del	0%	12%	NA
Chr 19 del	33%	9%	NA
Chr 7 amp	17%	17%	NA
4q12 amp	0%	10%	NA
12q13 amp	0%	4%	NA
12q15 amp	0%	4%	NA
17p13 del	0%	3%	NA
