# Supplementary material for: Genomic Profiling in Glioma Patients to Explore Clinically Relevant Markers
Source: Int J Mol Sci. 2024 Dec 3;25(23):13004. doi: 10.3390/ijms252313004 (PMC11641329; doi:10.3390/ijms252313004)
Supplement: Supplementary file 1 [file ijms-25-13004-s001.zip › Table S3. Median survival and 1-2-5-year survival.pdf]

Table S3. Median OS and 1-2-5 overall survival for different predictors.

Predictor		Median OS, month (95% CI)	1-year OS (95% CI)	2-year OS (95% CI)	5-year OS (95% CI)
Sex	Female	25.4 (15.7 – 34.9)	0.74 (0.63 – 0.87)	0.54 (0.41– 0.70)	0.09 (0.06– 0.32)
	Male	18.2 (14.6 – 29.3)	0.71 (0.59 – 0.85)	0.39 (0.27 – 0.55)	0.15 (0.07 – 0.32)
Cancer history	SPGB	9.3 (4.76 - NA)	0.29 (0.12 - 0.74)	0.20 (0.06 - 0.69)	-
	Only GB	22.2 (17.05 – 29.8)	0.78 (0.70 - 0.87)	0.49 (0.39 - 0.61)	0.11 (0.05 - 0.26)
Recurrence	One surgery resection	15.7 (11.8 - 29.8)	0.60 (0.49 - 0.73)	0.40 (0.29 - 0.54)	-
	Two or more resections	25.5 (19.8 – 44.4)	0.97 (0.92 - 1.00)	0.57 (0.43 - 0.77)	0.19 (0.08 - 0.44)
Tumor distribution	Multilobar	18.2 (11.4 – 38.0)	0.62 (0.48 - 0.81)	0.47 (0.32 - 0.69)	0.095 (0.026 - 0.35)
	Unilobar	19.8 (16.1 – 29.7)	0.78 (0.69 - 0.89)	0.46 (0.35- 0.60)	0.12 (0.05- 0.33)
Bevacizumab therapy	Non-received	12.3 (9.3 – 29.8)	0.52 (0.39 - 0.69)	0.36 (0.24 - 0.55)	0.13 (0.05 - 0.40)
	Received	25.5 (18.6 – 34.2)	0.88 (0.80 - 0.97)	0.53 (0.41 - 0.68)	0.11 (0.04 - 0.31)
MGMT promoter	Non-methylated	18.6 (15.2 – 29.7)	0.73 (0.61 - 0.90)	0.40 (0.27 - 0.59)	0.05 (0.01 - 0.29)
	Methylated	19.8 (14.6 – 49.1)	0.70 (0.57 - 0.86)	0.48 (0.34 - 0.67)	0.18 (0.07 - 0.48)
CDKN2A/B deletion	Wild-type	18.2 (15.6 – 29.7)	0.72 (0.62 - 0.84)	0.44 (0.33 - 0.58)	0.13 (0.06 - 0.30)
	Mutant	22.2 (15.2 – 38.0)	0.74 (0.61 - 0.90)	0.50 (0.35 – 0.70)	-
TERT promoter	Wild-type	29.3 (18.8 - NA)	0.82 (0.70 - 0.10)	0.59 (0.42 - 0.81)	0.26 (0.10 - 0.67)
	Mutant	17 (14.9 - 26)	0.69 (0.59 - 0.80)	0.41 (0.31 - 0.54)	0.06 (0.02 - 0.20)
PTEN deletion	Wild-type	26 (19.5 – 38.0)	0.86 (0.77 - 0.95)	0.56 (0.44 - 0.71)	0.10 (0.02 - 0.40)
	Mutant	15.1 (9.3 – 24.8)	0.55 (0.42- 0.72)	0.33 (0.21 - 0.51)	0.15 (0.06 - 0.38)
PTEN all alterations	Wild-type	27.1 (19.8 – 44.4)	0.89 (0.80 – 1.00)	0.60 (0.45 - 0.79)	0.11 (0.02 - 0.61)
	Mutant	16.1 (14.6 – 25.4)	0.63 (0.52 - 0.76)	0.38 (0.27 - 0.520	0.09 (0.03 - 0.28)
TP53	Wild-type	22.2 (15.6 – 31.8)	0.71 (0.62 - 0.83)	0.49 (0.39 - 0.62)	0.08 (0.02 - 0.35)
	Mutant	17 (14.9 – 29.3)	0.76 (0.62 - 0.93)	0.37 (0.22 - 0.61)	0.13 (0.04 - 0.44)
EGFR all alterations	Wild-type	24.8 (17.0 – 31.8)	0.79 (0.70 - 0.90)	0.51 (0.39 - 0.66)	0.16 (0.07 - 0.36)
	Mutant	15.2 (12.3 – 29.8)	0.64 (0.51 - 0.80)	0.39 (0.27 - 0.57)	-
Chromosome 7 gain	Wild-type	17.1 (14.6 – 29.8)	0.69 (0.58- 0.82)	0.41(0.30 - 0.57)	0.14 (0.05 - 0.37)
	Mutant	9.7 (7.85 - NA)	0.42 (0.20 - 0.88)	0.32 (0.12 - 0.80)	-
Chromosome 10 loss	Wild-type	26 (14.6 – 34.9)	0.76 (0.62 - 0.92)	0.51 (0.36 - 0.73)	-
	Mutant	14.9 (9.6 – 24.8)	0.57 (0.43 - 0.75)	0.31 (0.19 - 0.51)	0.21 (0.10 - 0.46)
