# Supplementary figures and images for: Genomic Profiling in Glioma Patients to Explore Clinically Relevant Markers
Source: Int J Mol Sci. 2024 Dec 3;25(23):13004. doi: 10.3390/ijms252313004 (PMC11641329; doi:10.3390/ijms252313004)

(a)

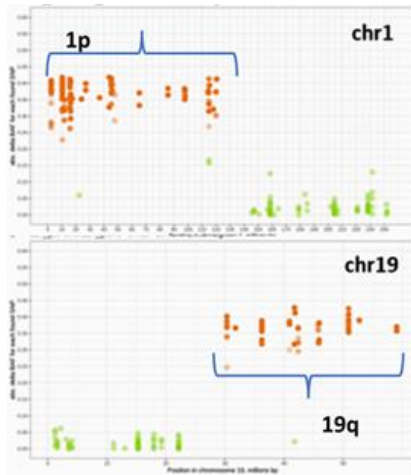

Co-deletion 1p/19q

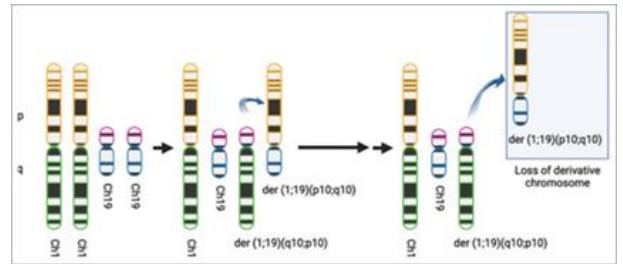

(b)

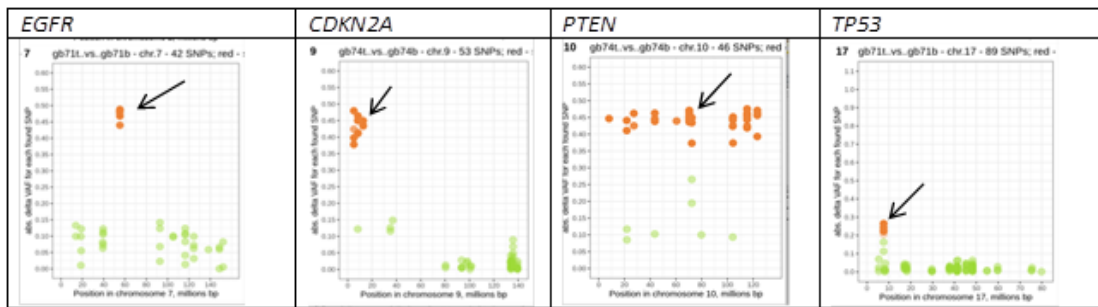

(c)

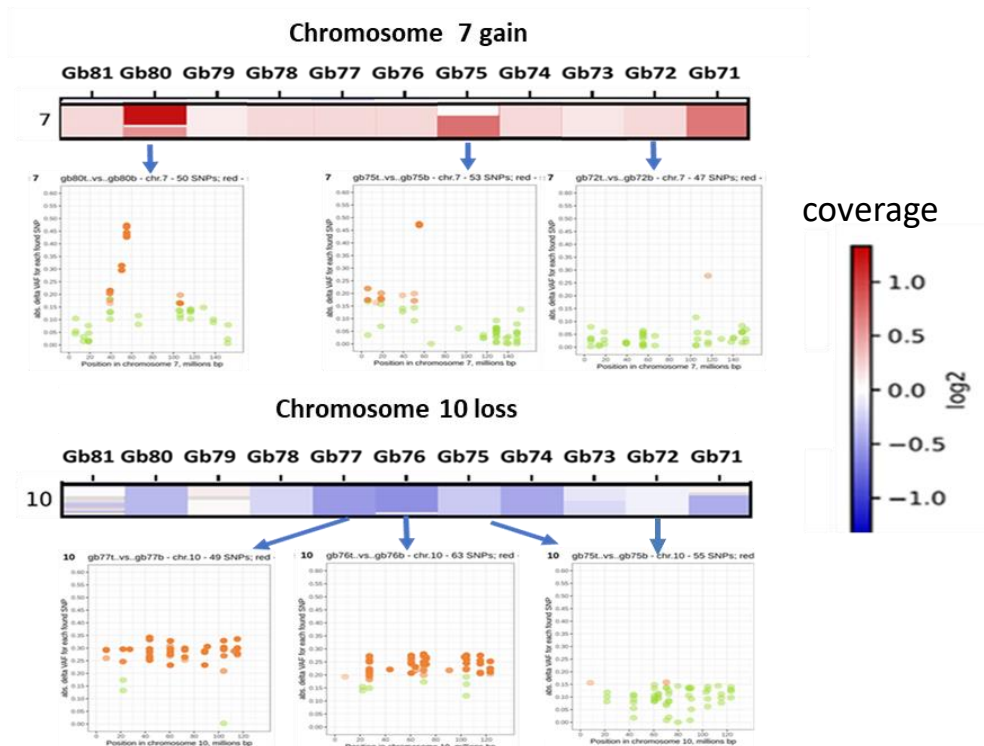

Figure S1. Examples of BAF-analysis

Supplement: Supplementary file 1 [file ijms-25-13004-s001.zip › Figure S1. BAF-analysis.pdf]
